# Surface modification of gold nanoparticles with neuron-targeted exosome for enhanced blood–brain barrier penetration

**DOI:** 10.1038/s41598-019-44569-6

**Published:** 2019-06-04

**Authors:** Mattaka Khongkow, Teerapong Yata, Suwimon Boonrungsiman, Uracha Rungsardthong Ruktanonchai, Duncan Graham, Katawut Namdee

**Affiliations:** 1 National Nanotechnology Centre (NANOTEC), National Science and Technology Development Agency, 111 Thailand Science Park, Paholyothin Rd., Klong Luang, Pathumthani, 12120 Thailand; 2 Centre for Molecular Nanometrology, Department of Pure and Applied Chemistry, Technology and Innovation Centre, University of Strathclyde, 99 George Street, G1 1RD Glasgow, United Kingdom

**Keywords:** Nanoscale biophysics, Membranes

## Abstract

Gold nanoparticles (AuNPs) have been extensively used as nanomaterials for theranostic applications due to their multifunctional characteristics in therapeutics, imaging, and surface modification. In this study, the unique functionalities of exosome-derived membranes were combined with synthetic AuNPs for targeted delivery to brain cells. Here, we report the surface modification of AuNPs with brain-targeted exosomes derived from genetically engineered mammalian cells by using the mechanical method or extrusion to create these novel nanomaterials. The unique targeting properties of the AuNPs after fabrication with the brain-targeted exosomes was demonstrated by their binding to brain cells under laminar flow conditions as well as their enhanced transport across the blood brain barrier. In a further demonstration of their ability to target brain cells, *in vivo* bioluminescence imaging revealed that targeted-exosome coated AuNPs accumulated in the mouse brain after intravenous injection. The surface modification of synthetic AuNPs with the brain-targeted exosome demonstrated in this work represents a highly novel and effective strategy to provide efficient brain targeting and shows promise for the future in using modified AuNPs to penetrate the brain.

## Introduction

Nanotechnology-based delivery systems have proven to be a promising tool for drug delivery^1^. These nanomedicine platforms can be categorized as organic-based (e.g., liposomes, lipid nanoparticles, biodegradable polymeric nanoparticles), inorganic-based (e.g., gold nanoparticles, mesoporous silica nanoparticles, porous silicon, silicon nanowires, carbon nanotubes, and calcium phosphate), or a hybrid combination of the aforementioned^2^.

Gold nanoparticles (AuNPs) displays the multifunctional characteristics in therapeutics, imaging, and surface modification. Therefore, they have been extensively used as nanomaterials for imaging and drug delivery, so-called “theranostic (combined therapy and diagnosis) system^3^”.

Similar to many other synthetic nanoparticle-based delivery systems, AuNPs have low specificity due to the lack of a selective moiety that can discriminate between targeted and non-targeted cells^4^. Currently, AuNPs have been combined with cell-targeting ligands to deliver therapeutic agents to targeted cells or tissues. Their large surface area provides a platform for conjugating multiple ligands (peptides, proteins, antibodies, or aptamers) as well as providing tunable nanomaterial properties such as porosity or optical responsiveness^5^.

Nevertheless, the development of the vast majority of actively-targeted AuNPs largely relies on synthetic conjugates using coupling chemistries to introduce functional moieties onto the surface of synthetic AuNPs^3^. These synthetic processes are complicated and usually very system-specific, which limits cross-system application. Furthermore, many materials are not suitable for clinical application due to poor biocompatibility and potential toxicity resulting from the use of inorganic materials or surfactants such as cetyl trimethylammonium bromide (CTAB)^6^.

Exosomes are natural nano-sized vesicles produced and secreted by cells. Recently, the exploitation of exosomes as a drug delivery system has received considerable attention and excitement^7^. Exosomes have been utilized as a drug delivery system for low molecular-weight drugs in several investigations^8–12^. Exosomes are non-immunogenic and non-toxic due to having a similar composition to the body’s cells. The exosome-based approach involves a simple, powerful and precise mechanism of biosynthesis and self-assembly^13^. Another point of contrast to the sophisticated and poorly controlled synthesis procedures used to incorporate peptides and antibodies to targeted vehicles is that exosomes can be genetically modified to improve delivery capacity and targeting specificity^14^.

In 2011, Alvarez-Erviti *et al*. first described exosome-mediated delivery of small interfering RNA (siRNA) to the mouse brain by intravenous injection. Exosome-producing cells were genetically engineered to express a fusion protein composed of the exosomal membrane protein Lamp2b and a neuron-targeted short peptide of rabies virus glycoprotein (RVG), allowing the engineered cells to produce and secret exosomes with the RVG peptide on their surface. These engineered exosomes could then selectively bind to the neuronal cells^15^. In a recent study, an improved version of the brain-targeted exosome was reported by engineering RVG peptide- Lamp2b fusion proteins to include a glycosylation motif in order to prevent peptide degradation and increase Lamp2b fusion protein expression in modified exosomes^13^.

To achieve high and efficient delivery of AuNPs to brain, surface modifications of AuNPs are needed, to add targeting moieties such as ligands, antibodies, and other directing agents to enhance transport across blood–brain barrier and selectively targeting into brain tissues^16–19^. This is significant because brain-targeted AuNPs system would be a promising platform and may have utility for facilitating the delivery of drugs and biological therapeutics (drug-loaded AuNPs) across blood-brain barrier for diagnosis and treatments of several central nervous system (CNS) disorders such as Alzheimer’s disease, Parkinson’s disease, brain cancers^20^.

**Figure 1:**
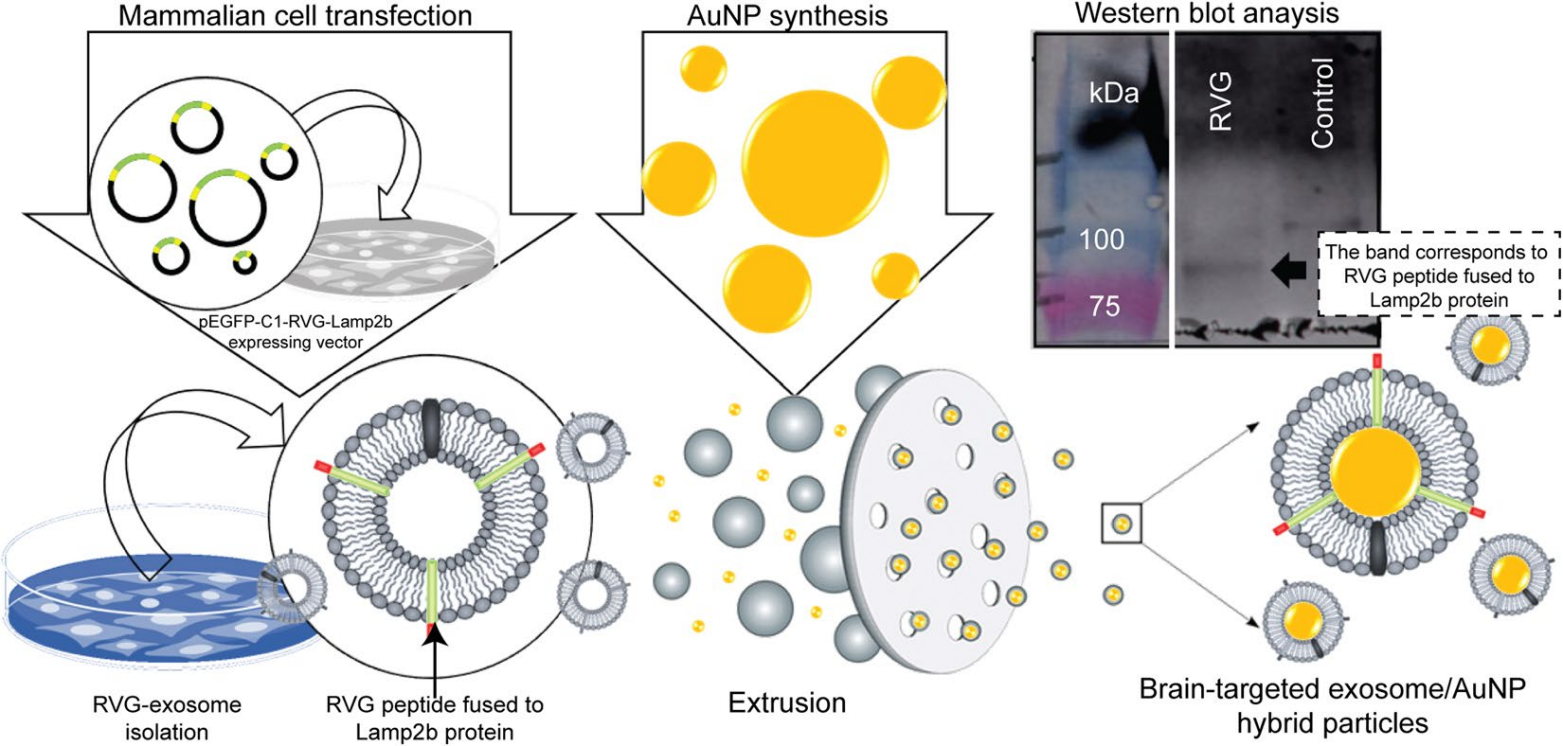
Schematic representation of gold nanoparticle surface functionalization through exosome coating. In this approach, exosome-producing cells were transiently transfected with a pcDNA GNSTM-3-RVG-10-Lamp2b-HA vector to express Lamp2b fusion protein fused in frame with the neuron-specific rabies viral glycoprotein (RVG) and glycosylation-stabilized (GNSTM) peptides, allowing the engineered cells to produce and secret exosomes with the RVG and GNSTM peptides on their surface as previously described by Hung, M. E., & Leonard, J. N., 2015^13^. These engineered exosomes were collected then coated onto the surfaces of synthetic nanoparticles using the mechanical method or extrusion. Western blot of exosomes produced from transfected or non-transfected cells and probed with anti-HA antibody against HA tag present in Lamp2b fusion protein are also shown in the figure.

Here we report the enhanced targeting properties of gold nanoparticles (AuNPs) encapsulated by the RVG-targeted exosomes previously isolated from the supernatant of genetically modified cell cultures. In order to confirm the expression of Lamp2b fusion protein, an aliquot of exosome preparations was also analysed by western blot. As shown in Fig. 1, the expression of Lamp2b fusion protein was detected in targeted exosomes but absent in exosomes produced from non-transfected cells. Representation of gold nanoparticle surface functionalization through exosome coating is schematically shown in Fig. 1. We show that through these modifications, synthetic AuNPs could be redesigned to specifically recognize and target neuronal cells and improve their transport across the blood brain barrier.

## Materials and Methods

### Generation of cells transiently expressing Lamp2b fusion protein

Sub-confluent monolayer cultures of transformed human embryonic kidneys (HEK293T) were cultured in serum free Dulbecco’s modified Eagle’s medium (DMEM) and transfected with pcDNA GNSTM-3-RVG-10-Lamp2b-HA vector for expression of Lamp2b fusion proteins in mammalian cells by lipofectamine (Invitrogen). Exosomes were harvested from the supernatant of transfected cells as previously described using serial centrifugation^13^. Brieftly, the supernatant was spun at 300 × *g* for 10 min, 2,000 × *g* for 10 min, and 12,000 × *g* for 30 min to remove cells, cell debris, and apoptotic bodies. Then, the supernatant were spun 120,000 × *g* for 120 min using ultracentrifuge (Beckman Coulter) to pellete exosomes. Exosome pellets were washed in PBS and pelleted again via ultracentrifugation before performing experiment. Exosomes also produced from non-tranfected cells were also prepared and used as control exosomes. Western blotting was performed for the analysis of Lamp2b fusion protein expression using the mouse monoclonal Anti-HA antibody (#H3663, Sigma). Primary antibody was detected with horseradish peroxidase-conjugated anti-mouse (# 62–6520, Invitrogen) immunoglobulin G secondary antibody.

### Exosome/AuNPs particles fabrication and characterization

Gold nanoparticles (AuNPs) were synthesized following Brown *et al*., 2010^21^. Briefly, NaAuCl_4_·2H_2_O (0.14 mmol) was dissolved in distilled water, and then the solution heated to 100 °C with continuous stirring. During boiling, sodium citrate (1% m/v) was added and boiled for 15 min before cooling to room temperature. UV-visible spectra (λ_max_) 519–523 nm was employed to indicate the successful formation of nanoparticles.

The AuNP coated exosome particles were fabricated by a serial extrusion method. Briefly, the harvested exosomes from serial centrifugation were mixed with 200 µL of AuNPs at Abs 1.0. The mixture ratio was estimated based on mean count rate of particles (DLS) and UV-VIS spectra of AuNPs. Both exosome and AuNPs were incubated together in 2 mL phosphate buffered saline (PBS) pH 7.4 at 4 °C for 30 min before extrusion. Consequently, the mixture of particles was then extruded serially through 400 nm, 200 nm and 100 nm polycarbonate porous membranes using a mini extruder. The particles were passed through the extruder repeatedly 10 times in each porous membrane to ensure that the AuNPs were entirely coated with exosome^22,23^. The mixture of particles was centrifuged at 8000 rpm for 20 min to purify the exosome/AuNPs from an excess of exosomes. The pellets were resuspended in 1 mL PBS, and the mean particle size measured before and after the exosome coating.

Nanoparticle size (nm), polydispersity and surface charge (zeta potential, mV) were measured by dynamic light scattering (DLS) using a Malvern XP. Moreover, the entire encapsulation of the exosome was observed by the Raman spectra of malachite green on AuNPs using Raman spectroscopy (Sierra Series Spectrometers (633 nm), Snowy Range Instruments, Laramie, WY, USA). In addition, the structure of the exosome/AuNPs was examined using a transmission electron microscope.

For fluorescence labeling, the nanoparticles were labeled with 1,1′-dioctadecyl-3,3,3′,3′-tetramethylindocarbocyanine perchlo-rate (DiI) dye. Dil labeling of exosome on nanoparticles, 1 mg/ml solution of Dil was added to PBS (1:750) and incubated for 30 min before removing excess dye by centrifugation. Dil-labeled particles were resuspended in PBS and centrifuged at 8,000 rpm for 20 min. This procedure was repeated for three times to wash excess dye away for further experiments.

### Nanoparticle binding efficiency

Immortalized bEnd.3 mouse brain endothelial cell line, C6 rat glioma cells, or Hela cervical cancer cells were cultured were cultured in Dulbecco’s modified Eagle’s medium (DMEM) with 10% fetal bovine serum (FBS), 3 mM L-glutamine and 1% penicillin–streptomycin. All cell thpes were maintained in a cell culture incubator at 37 °C and with 5% CO_2_. All cell lines were seeded in a 6-well plate till 90% of confluency over the bottom of wells prior to an experiment. The *in vitro* flow adhesion assay employed a parallel plate flow chamber (PPFC) equipped with silicon rubber gaskets forming the flow channel (GlycoTech, Gaithersburg, MD, USA). Modified or unmodified AuNPs in PBS buffer (pH 7.4) was then added and allowed to flow through the chamber as described in previously reported studies^24,25^. Briefly, a single straight flow channel (125 µm height) was placed over cultured cells and vacuum-seal to the flow deck in order to set up the flow chamber. The wall shear rate (*γ*_*w*_;WSR) in the system was calculated from the adjustment of volumetric flow rate (Q) according to

$${\gamma }_{w}({s}^{-1})=\frac{6Q}{w{h}^{2}}$$

where h is the height of channel (125 µm) and w is the channel width (1000 µm). The assays were conducted with laminar flow through the channel at WSRs of 100 s^−1^.

Nanoparticles were suspended in flow buffer (1%BSA in PBS) at a fixed concentration of 6.5 × 10^6^ particles/mL, and then introduced into the flow channel from an inlet reservoir at 100 ^s−1^ operated via a syringe pump (ProSence NE-1000, Oosterhout, Netherland). After 10 minutes, the system was washed by flow buffer and nanoparticle adhesion observed on an Olympus IX71 inverted microscope fitted with a digital camera (Olympus DP71). Particle binding density (#/mm^2^) was evaluated by manually counting of the number of bound particles on cells after 10 min of flow. This number was divided by the area of the field of view (20x magnification, A = 0.125 mm^2^) as described in a previous study^24^. The data was collected at a constant position along the length of the chamber for all experiments. Each data point represents an average of binding density from at least three independent experiments and includes at least 10 fields of view per experiment.

### Permeability of gold nanoparticles through blood brain barrier model (BBB model)

The experimental procedure for building up the *in vitro* BBB model is as according to Li *et al*., 2010^26^. First of all, the bottom side of the Transwell filters (0.4 m pore sizes, PET, 6 or 12 well; Corning, USA) were coated with collagen type I (8 g/cm^2^ in 0.02 N acetic acid) for 1 hr at room temperature (RT) to help cell attachment. Then, the filters were washed with PBS, and Astrocyte cells were seeded onto the back of the Transwell filters at a density of 2.44 × 10^4^ cells/cm^2^. After 3 h incubation, bEnd.3 cells were seeded onto the top side of the filters at a density of 10^5^ cells/cm^2^ and co-cultured with astrocyte cells for 3 days in DMEM with 10% fetal bovine serum, 3 mM glutamine, and 1% PS. The BBB models were characterized by detection of the tight junction proteins before performing experiments, and trans-epithelial electric resistance (TEER) was measured before and after treatments of nanoparticles.

Transwells were gently washed with PBS before adding nanoparticles stained with Dil in DMEM medium into the upper wells. The medium of lower wells were collected at 30 min, 1 hr, 3 hr, 6 hr, 12 hr and 24 hr time points. The concentration of permeated particles was determined by measurement of fluorescene intensity (SpectraMax). Permeability was evaluated in % as compared to total amount of nanoparticle added and remained in upper chamber.

### Murine model and *in vivo* assay

Methods were carried out in accordance with animal ethics guidelines. All experimental protocols were approved by the Animal Ethics Committee of Thammasat University [017/2560]. Fifteen C57BL/6wild type mice at 8–10 weeks old were used as animal models in this experiment. Mice were randomly assigned into three groups of five animals, and AuNPs/exosome were injected via the tail vein at a same dosage in 200 μL in PBS /mouse. 1 hour after injection, mice were anaesthetized with isoflurane. Then, mice were perfused with ice-cold PBS and 4% paraformaldehyde (PFA) in PBS, respectively. Mouse brains were harvested and snap-frozen in liquid nitrogen. Consequently, they were observed using an *in vivo* imaging instrument (Bruker). Subsequently, the brains were washed 3 times for 15 minutes with PBS before being soaked overnight with 20% sucrose. The brain was embedded in O.C.T. embedding medium and snap-frozen before cryostat sectioned (Leica CM1950).

### Statistical analysis

Significant differences between groups were analyzed by one-way or two-way analysis of variance (ANOVA) followed by Turkey’s multiple comparison post-tests. The statistical differences between two experimental groups were analyzed by Student’s unpaired t-test. p-values < 0.05 were considered significant.

## Results

In this study, we reported synthesis and physiochemical properties of gold nanoparticles (AuNPs) encapsulated by the RVG-targeted exosomes. Moreover, we demonstrated the enhanced targeting properties of this brain-targeted hybrid nanoparticle system *in vitro* as well as *in vivo* blood-brain barrier models.

### Physicochemical characteristics of the exosome-coated AuNPs

To gain an insight into the physicochemical properties of the exosome-coated AuNPs, we explored the surface chemistry of the AuNPs, exosomes and the hybrids. We first investigated the size characteristics of exosome-coated AuNPs by measuring their average diameter.

**Figure 2:**
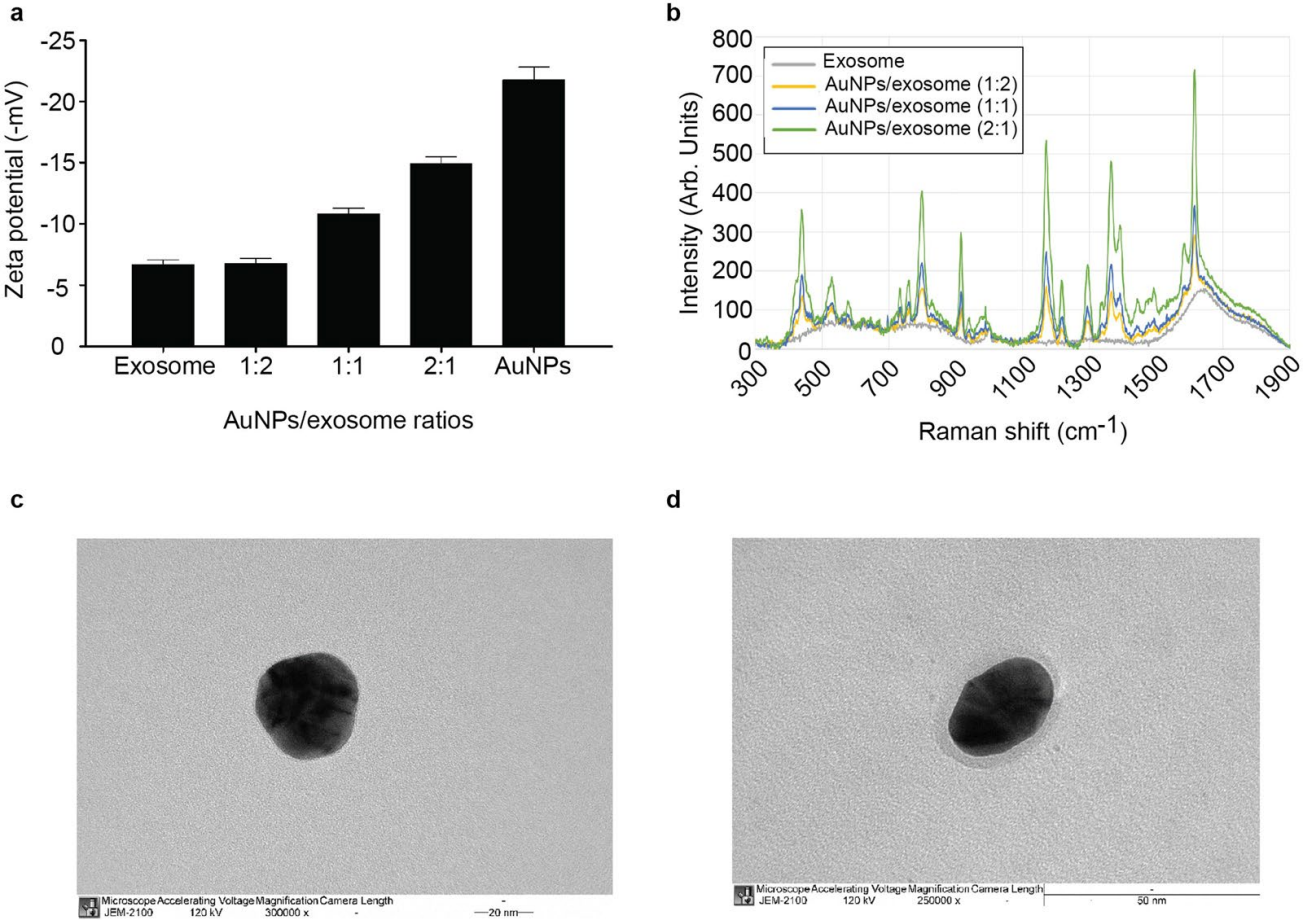
Physicochemical characteristics of the exosome-coated AuNPs. (**a**) The charge characteristics of exosome-coated AuNPs at different exosome/AuNPs ratios. (**b**) Raman spectra of exosome-coated AuNPs at different exosome/AuNPs ratios. (**c**) A representative TEM image of AuNP. (**d**) A representative TEM image of exosome-coated AuNP.

Subsequent experiments were performed to determine whether the surface charge characteristics of the hybrid nanoparticles is dependent on the amount of exosomes incorporated into the AuNPs at several AuNPs/exosome ratios. Different ratios of exosomes were found to have a significant effect on the ζ-potential values of the AuNP surfaces as presented in Figs 2a and S1. The addition of increasing amount of exosomes led to a corresponding decrease in the ζ-potential. For example, incorporation of exosomes resulted in a change in ζ-potential of AuNPs from −22 mV to −10 mV at an AuNP/exosome ratio of 1:1, after which the ζ-potential decreased to reach near the ζ-potential of the exosome at an AuNP/exosome ratio of 1:2. Therefore, the optimal ratio for the formation of exosome-coated AuNPs was determined to be 1:2 (AuNP/exosome).

Our result also showed that the Raman intensity of malachite green tagged AuNPs is dependent on the amount of AuNPs with an incomplete coating of exosome. As shown in Fig. 2b, the Raman shifts from a higher intensity for AuNPs, to a lower intensity for the hybrid nanoparticles following the addition of exosome and the extrusion process.

Having defined the optimal ratio that produced the hybrid nanoparticle to maintain the exosome ζ-potential, we analyzed the size, and the appearance of the exosome-coated AuNPs. Size measurement of the exosome-coated AuNPs in Table 1 revealed that the hybrids at optimal ratio of 1:2 (AuNP/exosome), had an average diameter correspondingly to the pore size of the polycarbonate membrane used in the extrusion. Formation of the exosome-coated AuNPs was confirmed by TEM. These images support the size results obtained by the NanoSizer. As shown in Fig. 2c,d, AuNPs were noticeably smaller than the exosome-coated AuNPs formed at an AuNP/exosome ratio of 1:2. TEM micrographs showed the spherical shape of the exosome-coated AuNP in which one gold nanoparticle (dark spot) was located in the center of the entire nanoparticle (brighter circle).

**Table 1:** Hydrodynamic size, polydispersity index, and ζ-potential.

Sample	Hydrodynamic size (d.nm)	PDI^a^
AuNPs	48 ± 1.5	0.247 ± 0.05
Exosome	225 ± 33.4	0.747 ± 0.14
AuNP/exosome	105 ± 10.1	0.430 ± 0.06

^a^PDI is Polydispersity Index.

The nanoparticles were either coated or uncoated with exosomes. The results are averages ±standard deviations (SD).

### The targeting property of AuNPs after fabrication with brain-targeted exosomes

**Figure 3:**
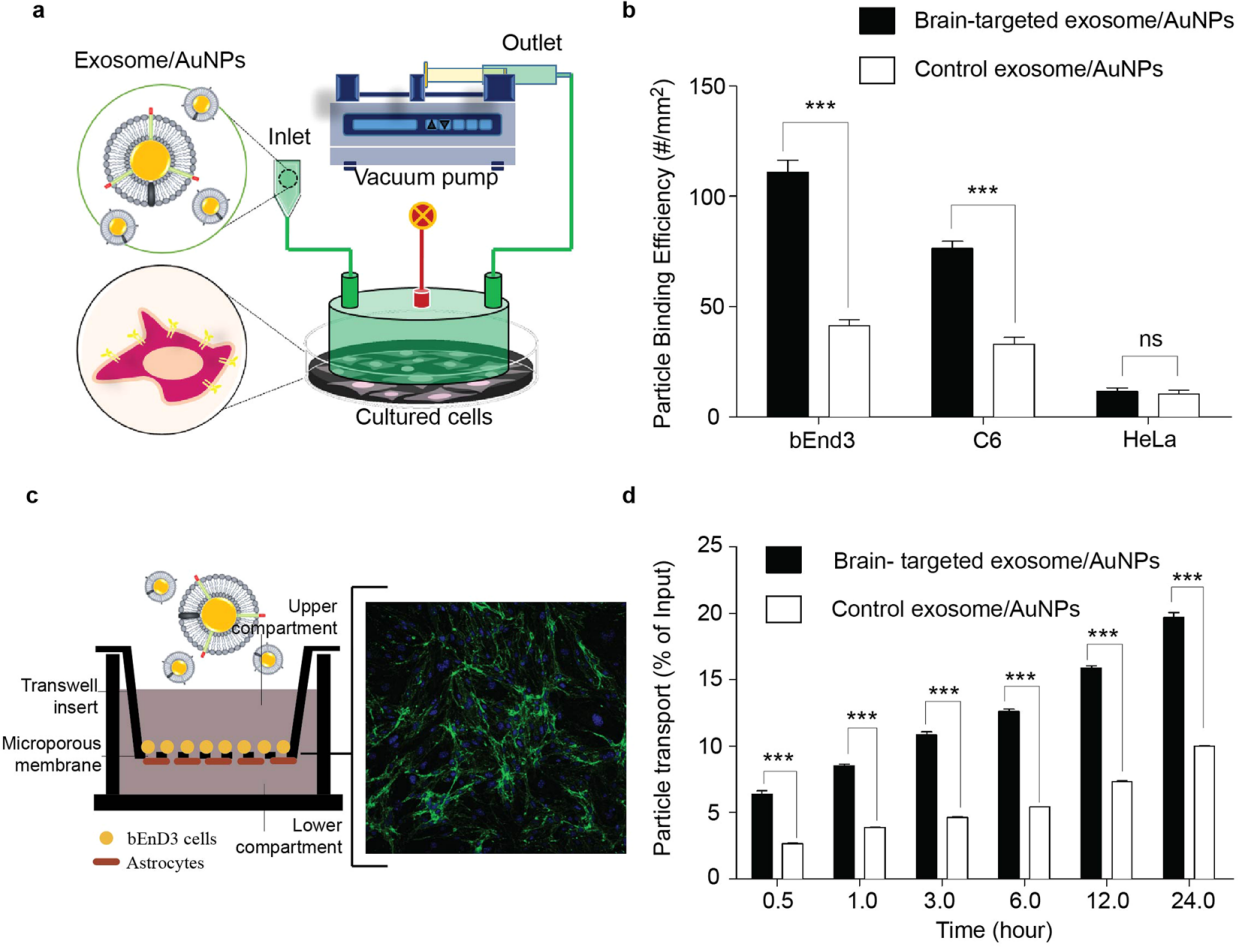
The brain-targeting property of AuNPs after fabrication with neuron-targeted exosome. (**a**) Schematic diagram showing the arrangement of the flow chamber perfusion system. The instrument required brain cells cultured on a tissue culture plate. A flow chamber was then assembled over the cultured cells and targeted exosome-coated AuNPs were flowed over the cultured cells. (**b**) Adhesion of nanoparticles to brain cells under flow conditions. Brain cells were exposed to targeted exosome-coated AuNPs or control AuNPs under flow condition. Hela cells were used as negative controls. As nanoparticles had been fluorescently labelled, the binding of nanoparticles to cells was visualized and analyzed using fluorescence microscopy. (**c**) An *in vitro* blood-brain barrier (BBB) model being composed of co-culture with endothelial (bEnd.3) and astrocyte-like (ALT) cells was established to evaluate the transcytosis of AuNPs coated with RVG- or unmodified exosomes. The expression of tight junction protein claudin-5 and ZO-1 in BBB model (Supplementary S2). Green: claudin-5 proteins; Blue: cell nucleus. (**d**) The percentage of exosome-coating AuNPs transported across the BBB over 20 hr. targeted exosome-coated AuNPs or control AuNPs were added to the apical chamber and incubated at 37 °C up to 24 hr. Signals of fluorescently labelled nanoparticles in the basal chamber were measured in 0.5 ml aliquots at different time points.

Following the hypothesis that the mechanism of enhanced brain cell specificity of the targeted AuNPs/exosome is associated with higher affinity to the cell surface than the non-targeted AuNPs/exosome, we carried out experiments to demonstrate the efficacy of modified AuNP adhesion to target cell membranes. The targeted exosome-coated AuNPs in flow buffer were larminarly flowed through PPFC and oberved from their binding to cultured cells on the bottom of chamber as a mimic of physiological conditions (as schematically shown in Fig. 3a).

The nanoparticles showed significantly higher targeting when the exosome-coated AuNPs were used compared with non-targeted nanoparticles to brain cells (Fig. 3b). Particularly, bEnd.3 and C6 had the highest binding (120 #/mm^2^). On the other hand (Fig. 3b), Hela had lower adhesion of targeted particles (20 #/mm^2^). The adhesion of nanoparticle were not different between active targeted and non-targeted exosome-coated AuNPs. These results suggest that the nanoparticles display specificity to only brain cells, which exhibit unique interaction to RVG exosome-coated AuNPs.

Next we investigated the effect of surface modification of AuNPs to cross the blood brain barrier by using a co-culture being composed of bEnd.3 brain endothelial cells and astrocyte-like (ALT) cells as an *in vitro* blood brain barrier model, as schematically shown in Fig. 3c. The trans-epithelial electric resistance (TEER) were measured in co-culture bEnd3/astrocytes and showed that a TEER increased up to approximately 164 Ω.cm^2^ prior to permeabilization study and remained consistent throughout the experiment (Supplement S2). Exosomes were labelled with Dil, a fluorescent lipophilic cationic indocarbocyanine dye as detailed in the methods. The percentage of exosome-coated AuNPs that crossed the BBB co-culture model into the basal chamber of the Transwell™ system over a 20 hr. incubation period was assessed using a fluorescence spectrophotometer (Fig. 3d). The fluorescent signal detected in the basal chamber was directly related to the amount of fluorescently labelled nanoparticles which crossed the cell monolayer. Percentage transport of AuNPs coated with RVG-exosome at 37 ^o^C was about 5% at 30 min. However, the percentage transported increased considerably and reached a maximum of around 20% at 24 hr. Incubating the cells with AuNPs coated with unmodified exosome resulted in a significant lower % transport than that obtained by AuNPs coated with RVG-exosome.

### *In vivo* brain uptake of exosome-coated AuNPs after intravenous injection

**Figure 4:**
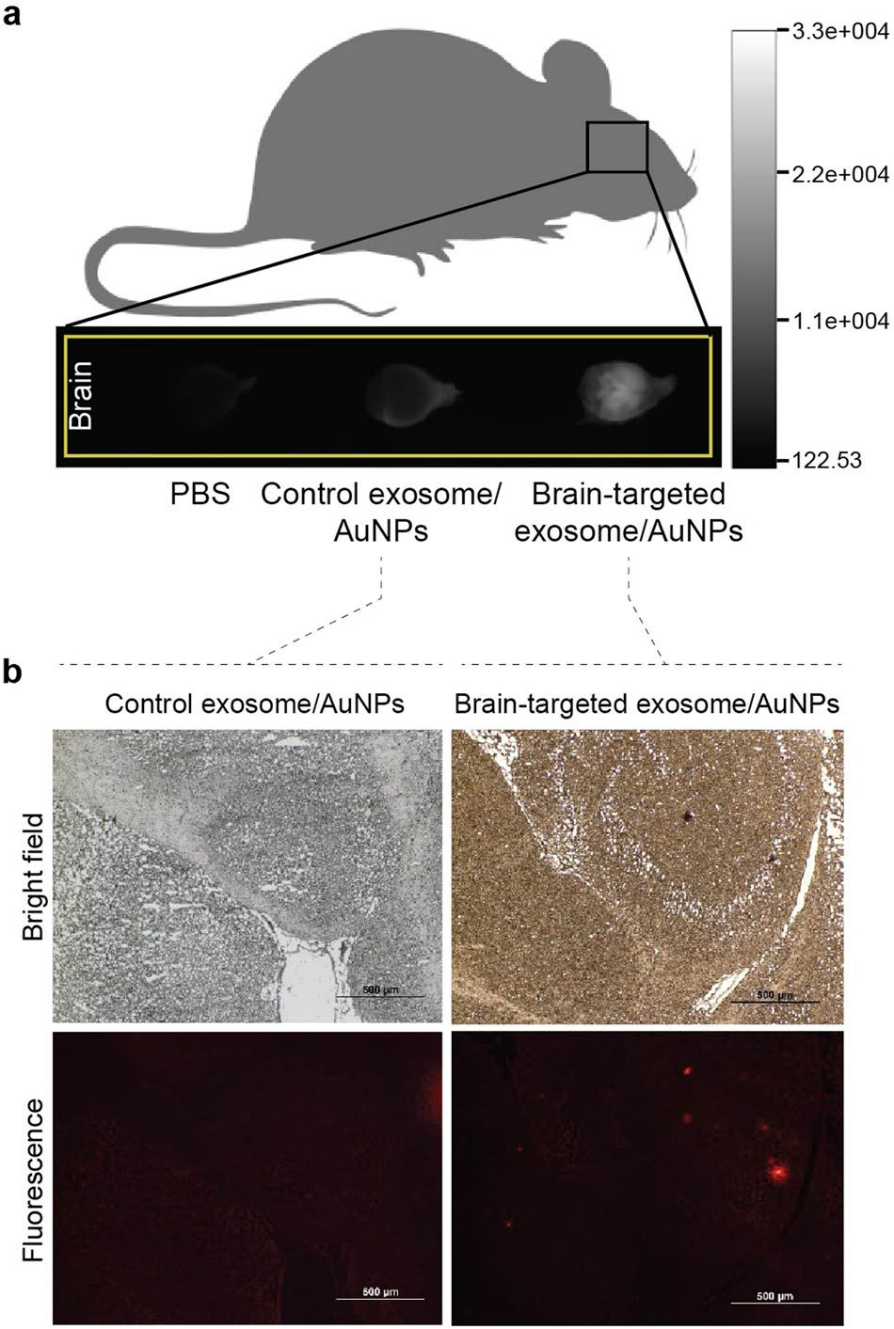
*In vivo* accumulation of exosome-coated AuNPs in brain tissue after intravenous injection. (**a**) Bioluminescence imaging of mouse brains following an intravenous injection of phosphate buffer saline (negative control), AuNPs coated with unmodified and RVG-exosomes (left to right). (**b**) AuNPs coated with unmodified and RVG-exosomes in mouse brain slices after an intravenous injection as examined by fluorescence microscopy.

Next, we examined the *in vivo* feasibility of exosome coated nanoparticles as a CNS delivery system using a murine model. AuNPs coated with RVG- or unmodified exosomes were administered to mice by tail vein injection. Uptake of fluorescently labelled nanoparticles into the brain was evaluated at 60 min post administration. The brain accumulation of the systems was assessed using a bioluminescence *in vivo* imaging system. As shown in Fig. 4a, no fluorescent signal was observed in the brains of negative control rats injected with phosphate buffer saline solution. Following injection of the AuNPs coated with exosomes, a signal was clearly visualized in the brain region. A higher brain uptake was observed following the administration of RVG-exosome coated AuNPs compared to the unmodified exosome coated AuNPs according to the signal intensity. The brain was also excised and sectioned. Fluorescent signals of brain sections were examined by fluorescence microscopy. Figure 4b represent images of the brain treated by the AuNPs coated with unmodified and RVG-exosomes, respectively. No or negligible fluorescence intensity was detected from brains of groups receiving AuNPs coated with unmodified. A strong signal was present in brains treated with AuNPs coated with RVG-exosomes.

## Discussion

Recently, a variety of drug-loaded nanoparticles (NPs) have been widely exploited to treat CNS disorders such as Alzheimer’s disease, Parkinson’s disease and brain cancers. In fact, synthetic nanoparticles have no intrinsic tropism for eukaryotic cells as they are chemically synthesized^27^. In order to develop a selective and efficient vector, it is therefore necessary to introduce novel tropism for brain targeting into these synthetic nanoparticles.

As aforementioned, AuNPs have gained considerable attention as promising theranostic agents for a variety of brain imaging and therapeutic applications^3,28–30^. This is significant because treatment and diagnosis for many neurological diseases are hindered by the inability of theranostic agents to cross the blood-brain barrier^29^. Even if a direct toxicity of AuNPs is minimal, increased brain accumulation of nanoparticles should be carried out for greater delivery of encapsulated theranostic agents at lower systemic doses^31^.

Adhesion to the cell surface is the first element that determines tissue tropism of the vector^32^. This process is necessary for the internalisation of vectors since the encapsulated particle must be transported from the cell surface to the intracellular compartment. The effectiveness of nanoparticles for targeted delivery to brain cells is limited partly due to the lack of targeting moieties recognized by associated cells. One strategy to enable transport of biopharmaceuticals into the brain is to utilize nanoparticles encapsulated with non-permeable molecules^33^. Specific rabies viral glycoprotein (RVG) peptide targeting the acetylcholine receptor (nAchR) attached to the surface of the nanoparticles allow their targeted delivery at the BBB^34^.

A previous investigation demonstrated that tropism for brain cells can be conferred on exosomes by fusing Lamp2b protein to a brain-targeting ligand, such as the neuron-specific rabies viral glycoprotein (RVG) peptide. In this respect, exosome-based vectors have been successfully adapted for brain targeting^15^. We examined brain cell binding of synthetic AuNPs after surface modification with neuron-targeted exosomes. Our results clearly indicate that the adhesion of modified AuNPs were significantly higher than the unmodified AuNPs.

Another major challenge is the blood brain barrier, which is formed by the endothelial cells that line cerebral microvessels if the target site is located in the central nervous system (CNS). Unlike blood capillaries elsewhere in the body, the structure of the BBB is characterized by the tight-junctions that are tightly resistant to the exchange of substances between the blood and the nervous tissue^35^. The transport of conventional systemic drugs into the CNS is therefore hindered partially due to the presence of blood brain barrier that acts as a physical and biochemical barrier^36^. In order to overcome this challenge, several strategies have been developed to enhance penetration of drugs into the brain. Of these, the most frequently used is receptor-mediated transcytosis. Gold nanoparticles (AuNPs) tagged with transferrin or L-DOPA is one of successful examples for brain targeting. The study of Cabezon, I. *et al*., 2015 reported that the physical modification of AuNPs with an external antibody against transferrin receptor has successfully achieved for brain internalization using ligand-receptor mediated vesicular fusion and rearrangement within few hours after exposure^37^. Similary, our results clearly demonstrate that the transport across BBB of AuNPs after genetically surface modification with RVG-exosome could be enhanced compared to AuNPs coated with unmodified exosomes. A bioluminescence signal was present in the excised brain after the administration of exosome-modified AuNPs, whereas it was not detectable after treatment with the aqueous solution. This result provides confirmation of the efficient passage of AuNPs fabricated with brain-targeting exosomes into the brain.

## Conclusion

In this study, we have developed and successfully tested an improved version of the hybrid vector concept by using neuron-targeted exosomes as coating materials for the delivery of gold nanoparticles across BBB to target brain cells. AuNP/RVG-exosome hybrid nanoparticles were successfully synthesized and investigated for their physiochemical and biological properties. AuNPs coated with surface-modified exosomes proved superior to those coated with unmodified exosome for specificity and transcytosis associated with brain cells. These results may accelerate the development of general design principles for surface modification of gold nanoparticles to cross the BBB, and help to meet the great challenge of providing treatments and imaging capabilities for brain diseases and disorders.

## Supplementary information

Supplementary info
