# Supplementary material for: Surface modification of gold nanoparticles with neuron-targeted exosome for enhanced blood–brain barrier penetration
Source: Sci Rep. 2019 Jun 4;9:8278. doi: 10.1038/s41598-019-44569-6 (PMC6547645; doi:10.1038/s41598-019-44569-6)
Supplement: Supplementary file 1 — Supplementary info [file 41598_2019_44569_MOESM1_ESM.docx]

**Supplementary Data**

**Title**

Surface modification of gold nanoparticles with neuron-targeted exosome for enhanced blood–brain barrier penetration

**Authors**

Mattaka Khongkow^1^, Teerapong Yata^1^, Suwimon Boonrungsiman^1^, Uracha Rungsardthong Ruktanonchai ^1^, Duncan Graham^2^ and Katawut Namdee^1,^*

**Authors’ affiliation**

^1^National Nanotechnology Centre (NANOTEC), National Science and Technology Development Agency, 111 Thailand Science Park, Paholyothin Rd., Klong Luang, Pathumthani 12120, Thailand.

^2^Centre for Molecular Nanometrology, Department of Pure and Applied Chemistry, Technology and Innovation Centre, University of Strathclyde, 99 George Street, G1 1RD Glasgow, United Kingdom.

^*^Author to whom correspondence should be addressed.

**Correspondence:**

Katawut Namdee*

National Nanotechnology Centre (NANOTEC), National Science and Technology Development Agency, Thailand.

Phone +66 2117 6500, Fax +66 2564 6985

E-Mail: [katawut@nanotec.or.th](mailto:katawut@nanotec.or.th)


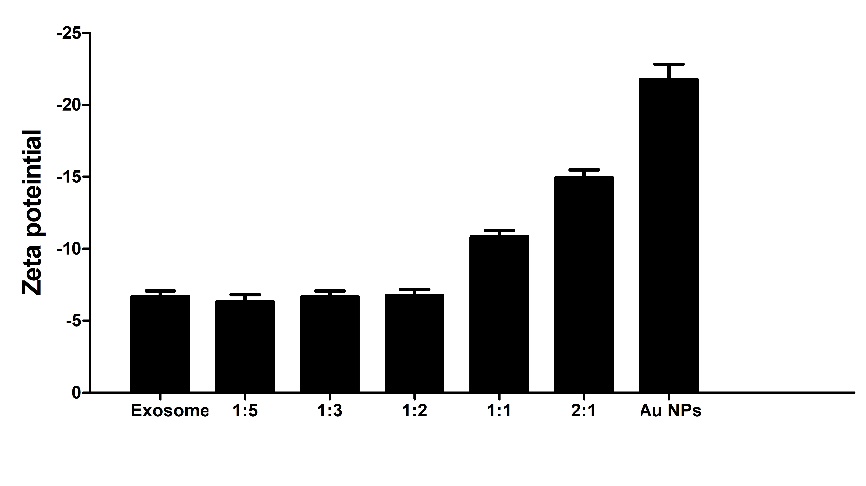


**Supplement 1 (S1).** The charge characteristics of exosome-coated AuNPs at different exosome/AuNPs ratios.

**ZO-1/DAPI**

**Cluadin-5/DAPI**


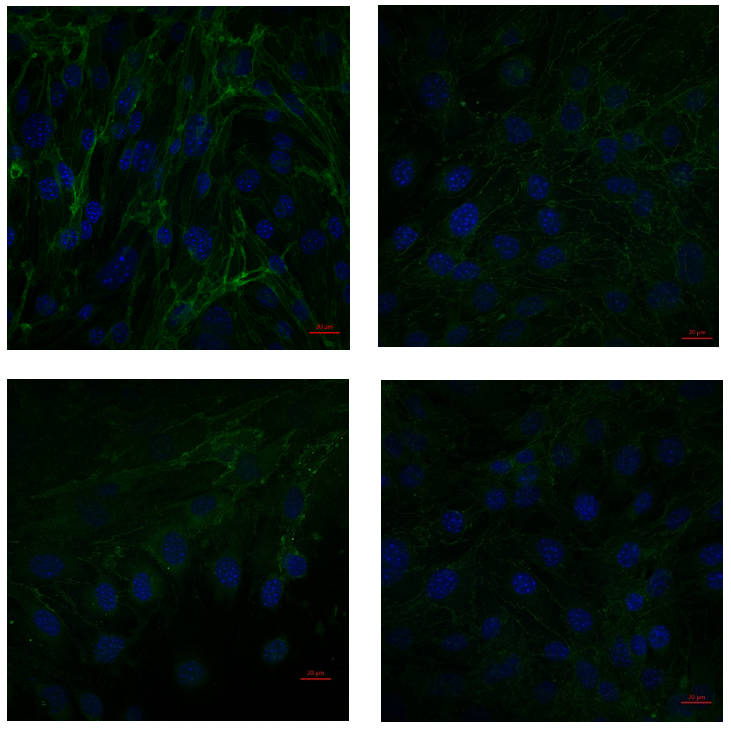


**bEnd3/Astrocyte**

**bEnd3**

**Supplement 2 (S2).** Immunofluorescence of tight junction expression in bEnd3/astrocytes co-culture model and bEnd3 monoculture by confocal microscope. Green (Alexa-Fluor 488): Cluadin-5 (352588, Invitrogen) and ZO-1 (ZO1-1A12, Invitrogen); Blue (DAPI, D1306, Invitrogen): cell nucleus. Scale bar = 20 μm. The trans-epithelial electric resistance (TEER) were measured in co-culture bEnd3/astrocytes and showed that a TEER increased up to approximately 164 Ω.cm^2^ prior to permeabilization study. The TEER remained consistent throughout the experiment and was around 169±3.5 Ω.cm2 at 24 hr. after


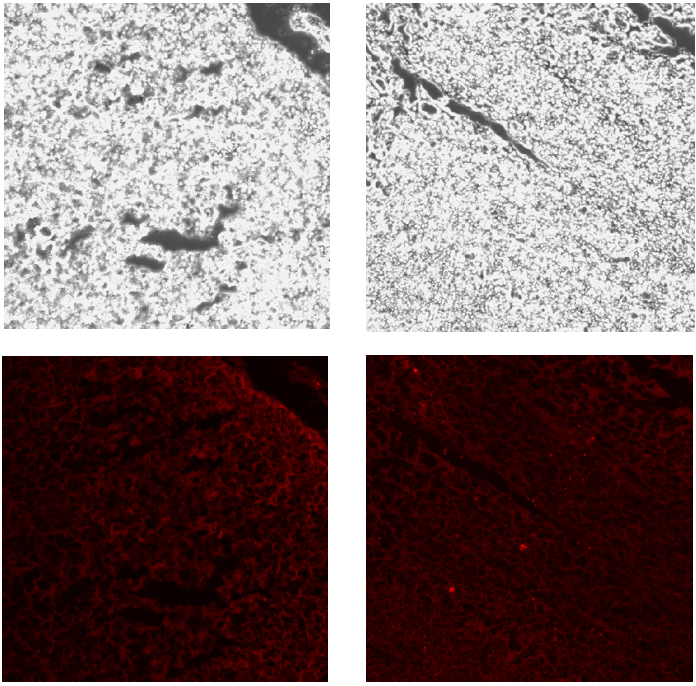


**Bright field**

**Fluorescence-Di**

Control exosome/AuNPs Brain-targeted exosome/AuNPs

**Supplement 3 (S3).** The fluorescence images (Dil) of nanoparticles coated with unmodified and RVG-exosomes in mouse brain slices after an intravenous injection as examined by confocal microscopy (scale bar = 100µm).


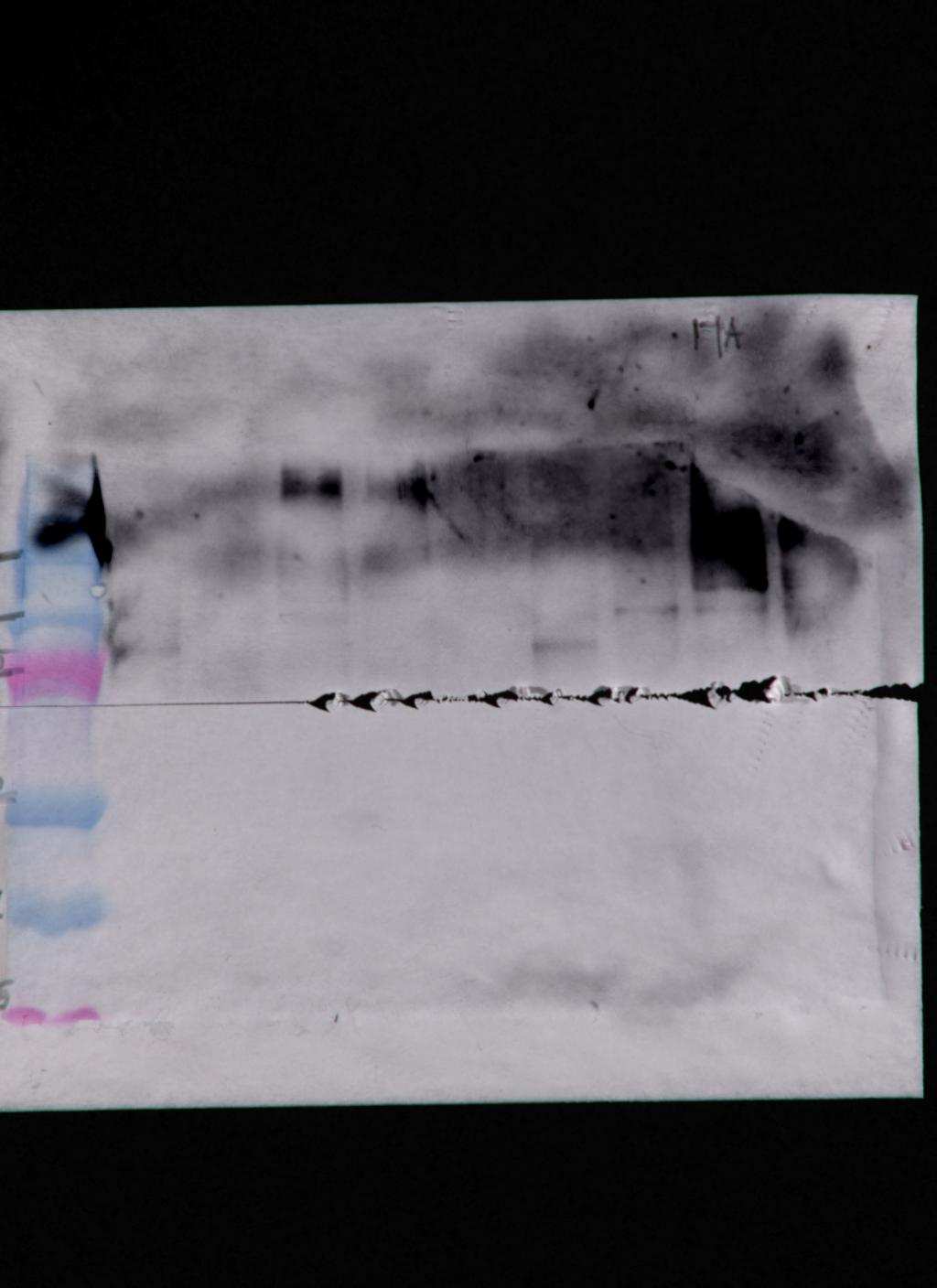

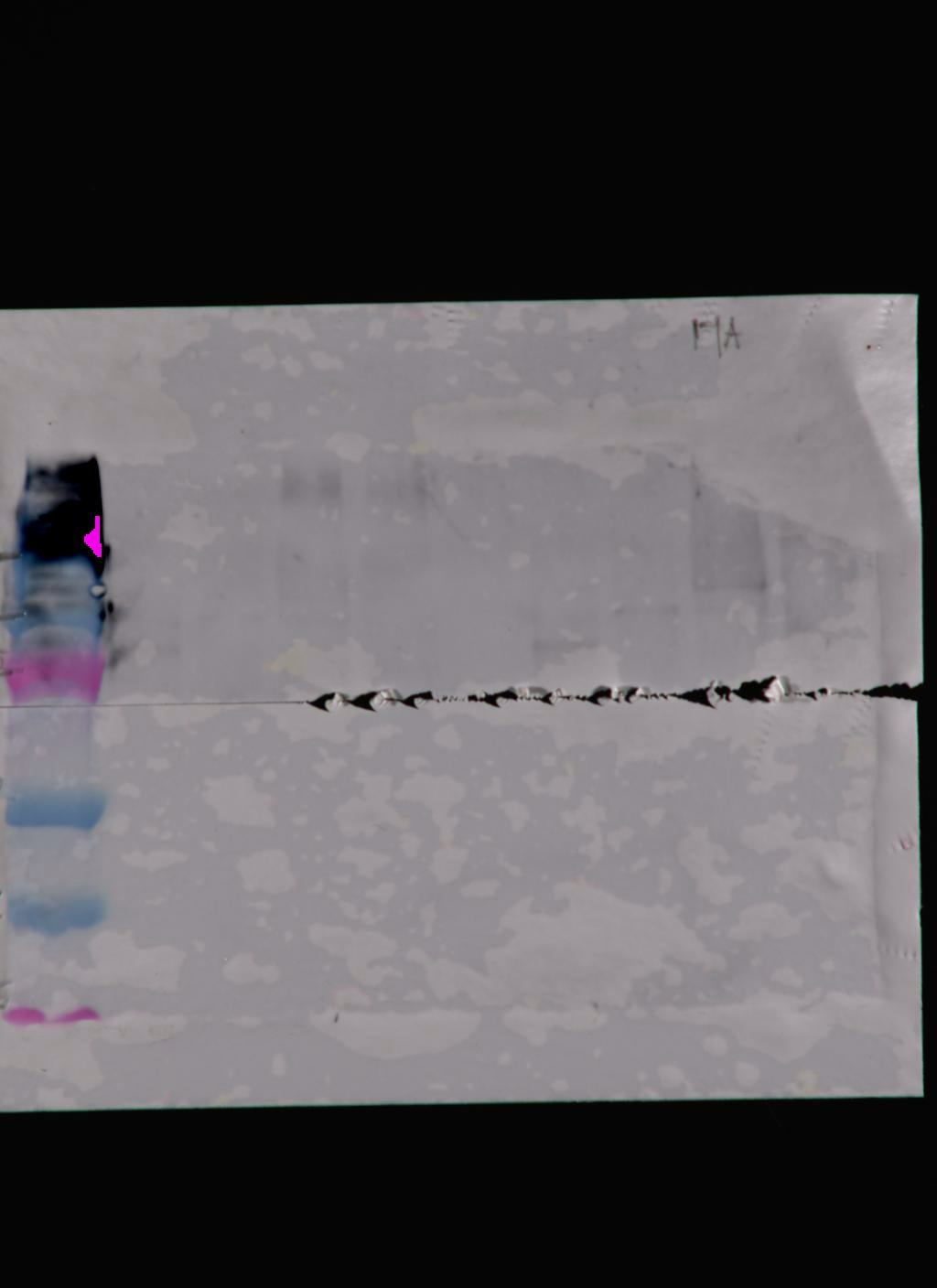


100

75

100

75

kDa

RVG

Control

Exosome

RVG fused Lamp2b-HA tag

Lower exposure

Cell

Exosome

Cell

RVG

Control

RVG

Control

RVG

Control

**Supplement 4**. Western blot of HEK293T cell lysates and exosomes produced from transfected or non-transfected HEK293T cells and probed with anti-HA antibody against HA tag present in Lamp2b fusion protein, showing that RVG fused Lamp2B proteins specifically express in produced exosomes but not in cell lysates.
